# 3-Chloro-*N*-(2-methyl­phen­yl)benzamide

**DOI:** 10.1107/S1600536811048756

**Published:** 2011-11-19

**Authors:** Vinola Z. Rodrigues, Peter Herich, B. Thimme Gowda, Jozef Kožíšek

**Affiliations:** a Department of Chemistry, Mangalore University, Mangalagangotri 574 199, Mangalore, India; b Institute of Physical Chemistry and Chemical Physics, Slovak University of Technology, Radlinského 9, SK-812 37 Bratislava, Slovak Republic

## Abstract

In the mol­ecular structure of the title compound, C_14_H_12_ClNO, the *meta*-Cl atom in the benzoyl ring is positioned *anti* to the C=O bond, while the *ortho*-methyl group in the aniline ring is positioned *syn* to the N—H bond. The two benzene rings are nearly coplanar [dihedral angle = 3.48 (5)°]. The crystal structure is stabilized by N—H⋯O hydrogen bonds, which link the mol­ecules into chains along the *b* axis.

## Related literature

For the preparation of the title compound, see: Gowda *et al.* (2003 ▶). For our studies on the effects of substituents on the structures and other aspects of *N*-(ar­yl)-amides, see: Bowes *et al.* (2003 ▶); Gowda *et al.* (2000 ▶); Rodrigues *et al.* (2011 ▶); Saeed *et al.* (2010 ▶), on *N*-(ar­yl)-methane­sulfonamides, see: Jayalakshmi & Gowda (2004 ▶) on *N*-(ar­yl)-aryl­sulfonamides, see: Shetty & Gowda (2005 ▶) and on *N*-chloro­aryl­amides, see: Gowda *et al.* (1996 ▶).
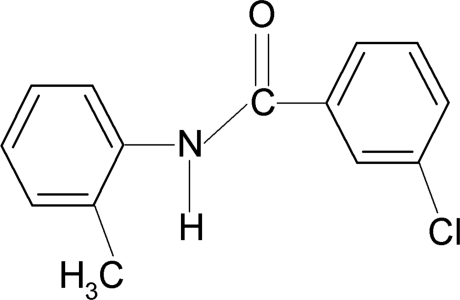


## Experimental

### 

#### Crystal data

C_14_H_12_ClNO
*M* *_r_* = 245.70
Monoclinic, 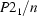

*a* = 11.1699 (5) Å
*b* = 4.9171 (2) Å
*c* = 21.4778 (8) Å
β = 90.339 (3)°
*V* = 1179.63 (8) Å^3^
*Z* = 4
Mo *K*α radiation
μ = 0.31 mm^−1^
*T* = 293 K
0.83 × 0.55 × 0.10 mm

#### Data collection

Oxford Diffraction Xcalibur Ruby Gemini diffractometer
Absorption correction: analytical [*CrysAlis RED* (Oxford Diffraction, 2009 ▶), based on expressions derived from Clark & Reid (1995 ▶)] *T*_min_ = 0.818, *T*_max_ = 0.970
22104 measured reflections
2411 independent reflections
2154 reflections with *I* > 2σ(*I*)
*R*_int_ = 0.050

#### Refinement

*R*[*F* ^2^ > 2σ(*F* ^2^)] = 0.036
*wR*(*F* ^2^) = 0.098
*S* = 1.04
2411 reflections
155 parameters
H-atom parameters constrained
Δρ_max_ = 0.18 e Å^−3^
Δρ_min_ = −0.24 e Å^−3^

### 

Data collection: *CrysAlis CCD* (Oxford Diffraction, 2009 ▶); cell refinement: *CrysAlis CCD*; data reduction: *CrysAlis RED* (Oxford Diffraction, 2009 ▶); program(s) used to solve structure: *SHELXS97* (Sheldrick, 2008 ▶); program(s) used to refine structure: *SHELXL97* (Sheldrick, 2008 ▶); molecular graphics: *DIAMOND* (Brandenburg, 2002 ▶); software used to prepare material for publication: *enCIFer* (Allen *et al.*, 2004 ▶).

## Supplementary Material

Crystal structure: contains datablock(s) I, global. DOI: 10.1107/S1600536811048756/bq2320sup1.cif

Structure factors: contains datablock(s) I. DOI: 10.1107/S1600536811048756/bq2320Isup2.hkl

Supplementary material file. DOI: 10.1107/S1600536811048756/bq2320Isup3.cml

Additional supplementary materials:  crystallographic information; 3D view; checkCIF report

## Figures and Tables

**Table 1:** Hydrogen-bond geometry (Å, °)

*D*—H⋯*A*	*D*—H	H⋯*A*	*D*⋯*A*	*D*—H⋯*A*
N1—H1*A*⋯O1^i^	0.86	2.11	2.9237 (18)	158

Symmetry code: (i) 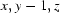.

**Fig. 1.**
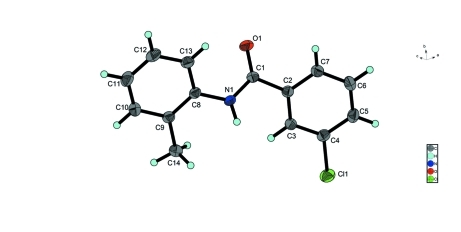
Molecular structure of the title compound showing the atom labeling scheme. Displacement ellipsoids are drawn at the 50% probability level.

**Fig. 2.**
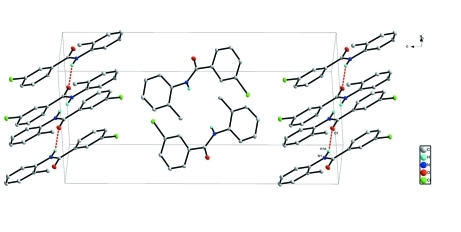
Part of the crystal structure of the title compound. Molecular chains are generated by N—H···O hydrogen bonds which are shown by dashed lines.

## supplementary crystallographic information

### Comment

The amide and sulfonamide moieties are the constituents of many biologically significant compounds. As part of our studies on the substituent effects on the structures and other aspects of *N*-(aryl)-amides (Bowes *et al.*, 2003; Gowda *et al.*, 2000; Rodrigues *et al.*, 2011; Saeed *et al.*, 2010), *N*-(aryl)-methanesulfonamides (Jayalakshmi & Gowda, 2004), *N*-(aryl)-arylsulfonamides (Shetty & Gowda, 2005) and *N*-chloro-arylamides (Gowda *et al.*, 1996), in the present work, the crystal structure of 3-Chloro-*N*-(2-methylphenyl)- benzamide (I) has been determined (Fig.1).

In (I), the N—H and C=O bonds in the C—NH—C(O)—C segment are *anti* to each other. The *meta*-Cl atom in the benzoyl ring is positioned *anti* to the C=O bond, while the *ortho*-methyl group in the anilino ring is positioned *syn* to the N—H bond, in contrast to the *syn* conformation observed between the *meta*-Cl atom in the benzoyl ring and the C=O bond in 3-Chloro-*N*-(3-methylphenyl)benzamide (II) (Rodrigues *et al.*, 2011), while the *meta*-methyl group in the anilino ring is positioned *anti* to the N—H bond. Further, the two aromatic rings are nearly coplanar with the dihedral angle of 3.48 (5)°, compared to the value of 77.4 (1)° in (II).

In the crystal structure, intermolecular N—H···O hydrogen bonds link the molecules into infinite chains running along the *b*-axis. Part of the crystal structure is shown in Fig. 2.

### Experimental

The title compound was prepared according to the method described by Gowda *et al.* (2003). The purity of the compound was checked by determining its melting point. It was characterized by recording its infrared and NMR spectra.

Rod like colorless single crystals of the title compound used in x-ray diffraction studies were obtained by slow evaporation of an ethanol solution of the compound (0.5 g in about 30 ml of ethanol) at room temperature.

### Refinement

All H atoms were visible in difference maps and then treated as riding atoms with C–H distances of 0.93 Å (C-aromatic), 0.96 Å (*C*-methyl) and N—H = 0.86 Å. The *U*_iso_(H) values were set at 1.2 *U*_eq_(C-aromatic, N) and 1.5 *U*_eq_(*C*-methyl).

### Crystal data

**Table:**

C_14_H_12_ClNO	*F*(000) = 512
*M**_r_* = 245.70	*D*_x_ = 1.383 Mg m^−^^3^
Monoclinic, *P*2_1_/*n*	Mo *K*α radiation, λ = 0.71073 Å
Hall symbol: -P 2yn	Cell parameters from 7179 reflections
*a* = 11.1699 (5) Å	θ = 3.4–26.4°
*b* = 4.9171 (2) Å	µ = 0.31 mm^−^^1^
*c* = 21.4778 (8) Å	*T* = 293 K
β = 90.339 (3)°	Rod, colorless
*V* = 1179.63 (8) Å^3^	0.83 × 0.55 × 0.10 mm
*Z* = 4	

### Data collection

**Table:**

Oxford Diffraction Xcalibur Ruby Gemini diffractometer	2411 independent reflections
Radiation source: Enhance (Mo) X-ray Source	2154 reflections with *I* > 2σ(*I*)
graphite	*R*_int_ = 0.050
Detector resolution: 10.4340 pixels mm^-1^	θ_max_ = 26.4°, θ_min_ = 3.4°
ω scans	*h* = −13→13
Absorption correction: analytical [*CrysAlis RED* (Oxford Diffraction, 2009), based on expressions derived from Clark & Reid (1995)]	*k* = −6→6
*T*_min_ = 0.818, *T*_max_ = 0.970	*l* = −26→26
22104 measured reflections	

### Refinement

**Table:**

Refinement on *F*^2^	Primary atom site location: structure-invariant direct methods
Least-squares matrix: full	Secondary atom site location: difference Fourier map
*R*[*F*^2^ > 2σ(*F*^2^)] = 0.036	Hydrogen site location: inferred from neighbouring sites
*wR*(*F*^2^) = 0.098	H-atom parameters constrained
*S* = 1.04	*w* = 1/[σ^2^(*F*_o_^2^) + (0.0481*P*)^2^ + 0.4496*P*] where *P* = (*F*_o_^2^ + 2*F*_c_^2^)/3
2411 reflections	(Δ/σ)_max_ < 0.001
155 parameters	Δρ_max_ = 0.18 e Å^−^^3^
0 restraints	Δρ_min_ = −0.24 e Å^−^^3^

### Special details

**Table:**

Experimental. CrysAlis RED (Oxford Diffraction, 2009) Analytical numeric absorption correction using a multifaceted crystal model based on expressions derived (Clark & Reid, 1995).
Geometry. All e.s.d.'s (except the e.s.d. in the dihedral angle between two l.s. planes) are estimated using the full covariance matrix. The cell e.s.d.'s are taken into account individually in the estimation of e.s.d.'s in distances, angles and torsion angles; correlations between e.s.d.'s in cell parameters are only used when they are defined by crystal symmetry. An approximate (isotropic) treatment of cell e.s.d.'s is used for estimating e.s.d.'s involving l.s. planes.
Refinement. Refinement of *F*^2^ against ALL reflections. The weighted *R*-factor *wR* and goodness of fit *S* are based on *F*^2^, conventional *R*-factors *R* are based on *F*, with *F* set to zero for negative *F*^2^. The threshold expression of *F*^2^ > σ(*F*^2^) is used only for calculating *R*-factors(gt) *etc*. and is not relevant to the choice of reflections for refinement. *R*-factors based on *F*^2^ are statistically about twice as large as those based on *F*, and *R*- factors based on ALL data will be even larger.

### Fractional atomic coordinates and isotropic or equivalent isotropic displacement parameters (Å^2^)

**Table:**

	*x*	*y*	*z*	*U*_iso_*/*U*_eq_	
C1	0.78713 (15)	0.6810 (3)	0.51026 (8)	0.0347 (4)	
C2	0.84225 (16)	0.5801 (3)	0.45112 (8)	0.0342 (4)	
C3	0.78692 (16)	0.3823 (3)	0.41438 (8)	0.0357 (4)	
H3A	0.7150	0.3041	0.4267	0.043*	
C4	0.84060 (17)	0.3043 (3)	0.35942 (8)	0.0380 (4)	
C5	0.94821 (18)	0.4136 (4)	0.34075 (9)	0.0434 (4)	
H5A	0.9839	0.3563	0.3040	0.052*	
C6	1.00224 (17)	0.6092 (4)	0.37740 (9)	0.0452 (4)	
H6A	1.0750	0.6837	0.3652	0.054*	
C7	0.94945 (17)	0.6957 (4)	0.43205 (8)	0.0395 (4)	
H7A	0.9856	0.8308	0.4559	0.047*	
C8	0.66916 (16)	0.5439 (3)	0.60171 (8)	0.0333 (4)	
C9	0.56240 (16)	0.4044 (3)	0.61282 (8)	0.0362 (4)	
C10	0.50367 (19)	0.4563 (4)	0.66843 (9)	0.0483 (5)	
H10A	0.4327	0.3650	0.6769	0.058*	
C11	0.5475 (2)	0.6388 (5)	0.71132 (9)	0.0547 (5)	
H11A	0.5059	0.6712	0.7479	0.066*	
C12	0.6531 (2)	0.7737 (4)	0.69999 (9)	0.0513 (5)	
H12A	0.6831	0.8967	0.7290	0.062*	
C13	0.71473 (18)	0.7262 (4)	0.64539 (8)	0.0418 (4)	
H13A	0.7865	0.8159	0.6379	0.050*	
C14	0.51064 (18)	0.2054 (4)	0.56700 (9)	0.0446 (4)	
H14C	0.4336	0.1460	0.5811	0.053*	
H14B	0.5630	0.0515	0.5635	0.053*	
H14A	0.5022	0.2914	0.5271	0.053*	
N1	0.73102 (13)	0.4923 (3)	0.54513 (6)	0.0348 (3)	
H1A	0.7328	0.3269	0.5321	0.042*	
O1	0.79372 (14)	0.9221 (2)	0.52456 (7)	0.0504 (4)	
Cl1	0.77036 (5)	0.06380 (10)	0.31224 (2)	0.05338 (17)	

### Atomic displacement parameters (Å^2^)

**Table:**

	*U*^11^	*U*^22^	*U*^33^	*U*^12^	*U*^13^	*U*^23^
C1	0.0383 (9)	0.0273 (8)	0.0386 (9)	0.0012 (7)	0.0004 (7)	−0.0005 (7)
C2	0.0385 (9)	0.0286 (8)	0.0356 (8)	0.0035 (7)	0.0020 (7)	0.0037 (6)
C3	0.0392 (9)	0.0319 (8)	0.0361 (8)	−0.0009 (7)	0.0040 (7)	0.0026 (7)
C4	0.0462 (10)	0.0328 (9)	0.0348 (9)	0.0036 (7)	−0.0019 (7)	0.0011 (7)
C5	0.0481 (10)	0.0468 (11)	0.0353 (9)	0.0062 (8)	0.0088 (8)	0.0017 (8)
C6	0.0383 (10)	0.0503 (11)	0.0469 (10)	−0.0025 (8)	0.0074 (8)	0.0069 (9)
C7	0.0415 (9)	0.0370 (9)	0.0401 (9)	−0.0023 (7)	0.0002 (7)	0.0013 (7)
C8	0.0386 (9)	0.0278 (8)	0.0336 (8)	0.0039 (7)	0.0019 (7)	−0.0005 (6)
C9	0.0406 (9)	0.0313 (8)	0.0369 (9)	0.0024 (7)	0.0002 (7)	−0.0004 (7)
C10	0.0485 (11)	0.0481 (11)	0.0484 (11)	−0.0019 (9)	0.0127 (9)	−0.0004 (9)
C11	0.0669 (14)	0.0582 (12)	0.0393 (10)	0.0033 (11)	0.0142 (9)	−0.0078 (9)
C12	0.0666 (13)	0.0481 (11)	0.0392 (10)	0.0016 (10)	−0.0026 (9)	−0.0138 (8)
C13	0.0449 (10)	0.0374 (9)	0.0429 (10)	−0.0016 (8)	−0.0011 (8)	−0.0074 (8)
C14	0.0440 (10)	0.0418 (10)	0.0479 (10)	−0.0062 (8)	0.0002 (8)	−0.0041 (8)
N1	0.0439 (8)	0.0258 (6)	0.0348 (7)	0.0000 (6)	0.0058 (6)	−0.0047 (5)
O1	0.0717 (10)	0.0252 (6)	0.0547 (8)	−0.0019 (6)	0.0158 (7)	−0.0037 (5)
Cl1	0.0666 (3)	0.0502 (3)	0.0434 (3)	−0.0055 (2)	−0.0005 (2)	−0.0113 (2)

### Geometric parameters (Å, °)

**Table:**

C1—O1	1.227 (2)	C8—C9	1.398 (3)
C1—N1	1.349 (2)	C8—N1	1.424 (2)
C1—C2	1.499 (2)	C9—C10	1.390 (3)
C2—C7	1.389 (3)	C9—C14	1.501 (2)
C2—C3	1.395 (2)	C10—C11	1.374 (3)
C3—C4	1.381 (2)	C10—H10A	0.9300
C3—H3A	0.9300	C11—C12	1.376 (3)
C4—C5	1.378 (3)	C11—H11A	0.9300
C4—Cl1	1.7412 (18)	C12—C13	1.383 (3)
C5—C6	1.380 (3)	C12—H12A	0.9300
C5—H5A	0.9300	C13—H13A	0.9300
C6—C7	1.384 (3)	C14—H14C	0.9600
C6—H6A	0.9300	C14—H14B	0.9600
C7—H7A	0.9300	C14—H14A	0.9600
C8—C13	1.392 (2)	N1—H1A	0.8600
			
O1—C1—N1	123.60 (16)	C10—C9—C8	117.66 (17)
O1—C1—C2	120.50 (16)	C10—C9—C14	120.08 (17)
N1—C1—C2	115.91 (14)	C8—C9—C14	122.26 (16)
C7—C2—C3	119.89 (16)	C11—C10—C9	121.86 (19)
C7—C2—C1	118.25 (16)	C11—C10—H10A	119.1
C3—C2—C1	121.82 (16)	C9—C10—H10A	119.1
C4—C3—C2	118.93 (17)	C10—C11—C12	119.91 (18)
C4—C3—H3A	120.5	C10—C11—H11A	120.0
C2—C3—H3A	120.5	C12—C11—H11A	120.0
C5—C4—C3	121.61 (17)	C11—C12—C13	119.99 (18)
C5—C4—Cl1	119.08 (14)	C11—C12—H12A	120.0
C3—C4—Cl1	119.31 (14)	C13—C12—H12A	120.0
C4—C5—C6	119.01 (17)	C12—C13—C8	119.91 (18)
C4—C5—H5A	120.5	C12—C13—H13A	120.0
C6—C5—H5A	120.5	C8—C13—H13A	120.0
C5—C6—C7	120.75 (18)	C9—C14—H14C	109.5
C5—C6—H6A	119.6	C9—C14—H14B	109.5
C7—C6—H6A	119.6	H14C—C14—H14B	109.5
C6—C7—C2	119.78 (17)	C9—C14—H14A	109.5
C6—C7—H7A	120.1	H14C—C14—H14A	109.5
C2—C7—H7A	120.1	H14B—C14—H14A	109.5
C13—C8—C9	120.67 (16)	C1—N1—C8	125.45 (14)
C13—C8—N1	120.84 (16)	C1—N1—H1A	117.3
C9—C8—N1	118.48 (15)	C8—N1—H1A	117.3
			
O1—C1—C2—C7	36.2 (2)	N1—C8—C9—C10	179.64 (16)
N1—C1—C2—C7	−144.13 (16)	C13—C8—C9—C14	−179.74 (17)
O1—C1—C2—C3	−141.61 (19)	N1—C8—C9—C14	−0.6 (3)
N1—C1—C2—C3	38.0 (2)	C8—C9—C10—C11	0.3 (3)
C7—C2—C3—C4	0.2 (3)	C14—C9—C10—C11	−179.4 (2)
C1—C2—C3—C4	177.98 (15)	C9—C10—C11—C12	−0.7 (3)
C2—C3—C4—C5	1.2 (3)	C10—C11—C12—C13	0.3 (3)
C2—C3—C4—Cl1	−178.37 (13)	C11—C12—C13—C8	0.6 (3)
C3—C4—C5—C6	−1.2 (3)	C9—C8—C13—C12	−1.0 (3)
Cl1—C4—C5—C6	178.37 (15)	N1—C8—C13—C12	179.91 (17)
C4—C5—C6—C7	−0.2 (3)	O1—C1—N1—C8	1.9 (3)
C5—C6—C7—C2	1.6 (3)	C2—C1—N1—C8	−177.71 (15)
C3—C2—C7—C6	−1.5 (3)	C13—C8—N1—C1	−40.0 (3)
C1—C2—C7—C6	−179.42 (16)	C9—C8—N1—C1	140.87 (17)
C13—C8—C9—C10	0.5 (3)		

### Hydrogen-bond geometry (Å, °)

**Table:**

*D*—H···*A*	*D*—H	H···*A*	*D*···*A*	*D*—H···*A*
N1—H1A···O1^i^	0.86	2.11	2.9237 (18)	158.

Symmetry codes: (i) *x*, *y*−1, *z*.

## References

[bb1] Allen, F. H., Johnson, O., Shields, G. P., Smith, B. R. & Towler, M. (2004). *J. Appl. Cryst.* **37**, 335–338.

[bb2] Bowes, K. F., Glidewell, C., Low, J. N., Skakle, J. M. S. & Wardell, J. L. (2003). *Acta Cryst.* C**59**, o1–o3. 10.1107/s0108270102019996 12506222

[bb3] Brandenburg, K. (2002). *DIAMOND* Crystal Impact GbR, Bonn, Germany.

[bb4] Clark, R. C. & Reid, J. S. (1995). *Acta Cryst.* A**51**, 887–897.

[bb5] Gowda, B. T., Dou, S. Q. & Weiss, A. (1996). *Z. Naturforsch. Teil A*, **51**, 627–636.

[bb6] Gowda, B. T., Jyothi, K., Paulus, H. & Fuess, H. (2003). *Z. Naturforsch. Teil A*, **58**, 225–230.

[bb7] Gowda, B. T., Paulus, H. & Fuess, H. (2000). *Z. Naturforsch. Teil A*, **55**, 791–800.

[bb8] Jayalakshmi, K. L. & Gowda, B. T. (2004). *Z. Naturforsch. Teil A*, **59**, 491–500.

[bb9] Oxford Diffraction (2009). *CrysAlis CCD* and *CrysAlis RED* Oxford Diffraction Ltd, Yarnton, England.

[bb10] Rodrigues, V. Z., Kucková, L., Gowda, B. T. & Kožíšek, J. (2011). *Acta Cryst.* E**67**, o3277. 10.1107/S1600536811047271 PMC3238934 22199783

[bb11] Saeed, A., Arshad, M. & Simpson, J. (2010). *Acta Cryst.* E**66**, o2808–o2809. 10.1107/S1600536810040262 PMC3008970 21589001

[bb12] Sheldrick, G. M. (2008). *Acta Cryst.* A**64**, 112–122. 10.1107/S0108767307043930 18156677

[bb13] Shetty, M. & Gowda, B. T. (2005). *Z. Naturforsch. Teil A*, **60**, 113–120.

